# A Cross-Sectional Study on the Evidence-Based Dentistry, Perception Basis, and Use of Articaine Among Dental Practitioners

**DOI:** 10.7759/cureus.32510

**Published:** 2022-12-14

**Authors:** Richa Shree, Monica Ritu Kedia, Toshi Toshi, Nikhil Raj, Kumar Anand, Niharika Shahi

**Affiliations:** 1 Department of Orthodontics and Dentofacial Orthopaedics, Buddha Institute of Dental Sciences and Hospital, Patna, IND; 2 Department of Periodontics, Buddha Institute of Dental Sciences and Hospital, Patna, IND; 3 Department of Conservative Dentistry and Endodontics, R. K. D. F. Dental College and Research Centre, Bhopal, IND; 4 Department of Oral Medicine and Radiology, Buddha Institute of Dental Sciences and Hospital, Patna, IND; 5 Department of Pedodontics and Preventive Dentistry, Purvanchal Institute of Dental Sciences, Gorakhpur, IND

**Keywords:** local anesthesia, evidence-based dentistry, dentistry, dental practitioners, articaine

## Abstract

*Background:* Many dentists use articaine as their choice of local anesthetic agent. However, the use of articaine is limited to inferior alveolar nerve blocks (IANBs), and literature data are scarce concerning its perception and choice among various dental practitioners.

*Aim:* The aim of the present study was to assess the extent of articaine use as a local anesthetic in dentistry, its perception basis, and the consistency of evidence of the efficacy and safety of articaine in recent practice.

*Methods:* The present study utilized a survey tool that was given to all the participating dental practitioners, and the participants were given five minutes to fill out the survey questionnaire. The questionnaire was in English and had 14 questions to be answered. The data gathered were statistically assessed to formulate the results.

*Results: *The results of this cross-sectional survey reported that articaine is used as a choice of local anesthetic by more than half of the participating dental practitioners. Sixty percent (n = 480) participants used articaine in their practice, followed by lidocaine, which was used by 33% (n = 264) dental practitioners, mepivacaine by 2% (n = 16) participants, prilocaine by 1% (n = 8) dental practitioners, and other local anesthetics by 4% (n = 32) participants, respectively. Concerning the safety and efficacy of articaine use, 43% used it for all procedures except for IANBs, whereas 20% used it for all procedures, including IANBs.

*Conclusion: *Despite the reported efficacy and safety of articaine as a local anesthetic for all dental procedures, many dental practitioners refrain from using it, especially for IANBs. These data depict a difference between current research evidence and reported clinical practice.

## Introduction

Articaine is a local anesthetic agent that is amide-based and used routinely during various dental procedures. It has been used by various dentists since its introduction to clinical practice in 1976. Before the introduction of articaine, lidocaine was the most commonly used local anesthetic agent globally. However, after its widespread use in 1995, literature data reported a high incidence of lingual nerve paresthesia after the use of articaine, establishing a correlation between the two [1]. Also, following these data, other studies in 2009 and 2010 reported similar findings of lingual nerve paresthesia after articaine use. However, these authors reported an association of lingual nerve paresthesia with nearly four percent of local anesthetic solutions. The reported incidence of lingual nerve paresthesia after articaine was reported to be one in 609,000 and one in 4,159,848 in the reviews conducted in 2009 and 2010, respectively [2].

Later, the safety and efficacy of articaine were confirmed by various studies conducted from 2010 onward to now. Most of these studies were meta-analyses and systematic reviews, which are considered to have the highest reliability. In all the literature data reported from 2005-2018 to the present, no data, including clinical studies and randomized controlled trials, have reported any permanent lingual nerve paresthesia after the use of articaine as a local anesthetic agent in dental procedures [3]. 

The study conducted in 2004 also reported that the use of articaine as a local anesthetic agent for dental procedures is efficacious and safe, with no reported major adverse effects [4]. These results were also supported by other studies by various authors reporting that articaine is safe and efficacious for dental procedures with no major adverse effects. Although the efficacy and safety of articaine allow it to be used as a local anesthetic for dental procedures, its use is still limited as various dental practitioners are still afraid of initial reports mentioning lingual nerve paresthesia with the clinical use of articaine. Also, biases have been associated with different studies that have led dentists to refrain from using articaine [4]. 

The literature is scarce concerning the perception and use of articaine by dental practitioners, especially while giving the inferior alveolar nerve block (IANB). One study survey was done on Australian dental practitioners in 2010 concerning their education, the reason for choosing articaine, and its use [5]. It showed that articaine was used by the majority of Australian dentists, and their choice was influenced by their peers using it, educational courses, and previous literature data. However, the study further showed that nearly one-third of dentists did not use articaine for the IANB but for all other dental procedures. In the comparison of the safety and efficacy of articaine to lidocaine, articaine was found to be equal in some studies and superior in a few others in terms of safety and efficacy compared to lidocaine [5].

This cross-sectional study was based on the Australian study to assess the perception of dental practitioners and evidence-based dentistry on using articaine as a local anesthetic agent in dentistry. This study was aimed at ascertaining if dentists use evidence-based dentistry in their practice. The present study first collected data from the survey questionnaire from different dental practitioners about articaine, their perception, use, and basis for its use. This was followed by analyzing the gathered data and assessing whether these results align with evidence of the efficacy and safety of articaine use in routine practice.

## Materials and methods

The present cross-sectional clinical study was carried out to assess the extent of articaine use as a local anesthetic in dentistry, its perception basis, and the consistency of evidence of efficacy and safety of articaine in recent practice. The study was carried out at the Buddha Institute of Dental Sciences and Hospital in Patna, Bihar, India after the Ethical Committee gave clearance to proceed with the study [BIDS/2022/203]. The study's participants were dental practitioners.

The tools used for the present study were survey questionnaires, where the samples were contributed by the dental practitioners, who were assessed for the perception and use of articaine as a local anesthetic agent in the dental practice based on their perceptions and experiences. The study assessed 800 dentists, and survey questionnaires were given to the study participants. The participants were given five minutes to respond to the questionnaire.

The survey questionnaire was given in English for ease of understanding. The survey comprised 14 questions that were to be answered in five minutes. Before enrolling the participants in the survey, informed consent was obtained in both verbal and written form. The survey questionnaire focused on assessing the proportion of dentists using articaine in their routine practice, their perception of the efficacy and safety of articaine use as a dental local anesthetic agent, and the factors that affect dental practitioners' perceptions of the use of articaine.

The survey collected the demographics of the participating dentists, the use of local anesthetic agents and their preference for local anesthesia (LA) use, articaine use in dentistry and for the IANB, and the views of the participating dentists on the safety and efficacy of the use of articaine, their perceptions of the use of articaine in routine dental practice, a comparison of articaine to lidocaine for efficacy and safety of the local anesthetic agent, and any adverse effects encountered following the IANB. When adverse effects were encountered with the IANB, the type of LA used and the nature of the adverse effects were also evaluated. Any change adopted in the practice after adverse effects were encountered was also evaluated through the survey. Additionally, if dentists wanted to share any information concerning the adverse effects of LA use, that information was also noted. The participants for the study were recruited from private practitioners, dental specialists, and teaching dentists working in dental colleges and institutions. For each group of dentists, the survey questions were modified as and when needed. The survey was presented to the respondents in online formats as well it was given physically, which was shared through the social media platform to increase the availability of the questionnaire to more dental practitioners than that could be reached in person.

Also, the availability of surveys is much higher in the online format, which increases the chances of getting more honest answers than in a personal meeting where identities are disclosed. Also, the online survey follows the norms of coronavirus disease 2019 protocols. The data were gathered statistically by use of the statistical package IBM SPSS Statistics for Windows, Version 23 (Released 2015; IBM Corp., Armonk, New York, United States) to get the results.

## Results

Eight hundred dental practitioner participants completed the survey and contributed to the study data. A total of 864 practitioners were approached, but 64 practitioners either left the survey incomplete or did not give consent to participate in the study which can be due to identification issues or network error in cases with the online survey. The mean age of the study participants was 32.6±4.82 years. The majority of the study participants were in the age range of 31-40 years with 40.12% (n=321) participants followed by 30.25% (n=242) participants in the age range of 21-30 years, 10.37% (n=83) participants in the age range of 51-60 years, 9.62% (n=77) subjects in the age range of 41-50 years, 8.5% (n=68) participants from ≤20 years of age, and least 1.12% (n=9) subjects in the age range of >60 years. There were 61.12% (n=489) male participants and 38.87% (n=311) female participants in the present study. Among participants, there were 86% (n=688) general dentists, 9% (n=72) specialists, 3% (n=24) undergraduate dental students, and 2% (n=16) postgraduate dental students. Among these participants, 69% (n=552) were private practitioners, 19% (n=152) were working in the public sector, 9% (n=72) participants were in the educational field, and 3% (n=24) participants worked in the research field (Table 1).

**Table 1:** Demographics of the study participants

S. No	Characteristics	Percentage (%)	Number (n=800)
1.	Mean age (years)	32.6±4.82	
2.	Age range (years)		
a)	≤20	8.5	68
b)	21-30	30.25	242
c)	31-40	40.12	321
d)	41-50	9.62	77
e)	51-60	10.37	83
f)	>60	1.12	9
3.	Gender		
a)	Male participants	61.12	489
b)	Female participants	38.87	311
4.	Local anesthesia preference		
a)	Articaine	60	480
b)	Lidocaine	33	264
c)	Mepivacaine	2	16
d)	Prilocaine	1	8
e)	Others	4	32
5.	Profession		
a)	General dentist	86	688
b)	Specialists	9	72
c)	Dental students (undergraduate)	3	24
d)	Dental students (postgraduate)	2	16
6.	Practice sector		
a)	Research	3	24
b)	Education	9	72
c)	Public	19	152
d)	Private	69	552

On assessing the use of articaine in dental practice, it was seen that 60% (n=480) participants used articaine in their practice followed by lidocaine used by 33% (n=264) dental practitioners, mepivacaine by 2% (n=16) participants, prilocaine by 1% (n=8) dental practitioners, and other local anesthetics by 4% (n=32) participants, respectively. Concerning the safety and efficacy of articaine use, 43% used articaine for all procedures except for the IANB, whereas 20% used it for all procedures including the IANB. Others did not use them in various other conditions including for absolute contraindications, pediatric subjects, and pregnant females. Forty-three percent of participants considered articaine to be more efficacious and safer compared to lidocaine, 34% considered it less safe but more efficacious than lidocaine, 19% found it to be equally efficacious and safe as lidocaine, and four participants considered articaine as unsafe to be used as a local anesthetic.

Among 800 study participants, 12.25% (n=98) participants reported one or other adverse effect following the use of a LA. The most common adverse effect was paresthesia reported by 30 participants where 11 cases were reported with lidocaine, 15 with articaine, and 2 each with mepivacaine and other local anesthetics. Neuropathy was reported in two cases with lidocaine, 4 with articaine, and 2 with other local anesthetic agents, trismus was reported in eight cases with lidocaine and 2 with other local anesthetic agents, hematoma was reported by four subjects with lidocaine and 2 with prilocaine, palsy was reported by four subjects with lidocaine and 2 with articaine, anxiety or palpitations was reported by 12 subjects with lidocaine and 4 with articaine, syncope was reported by two participants using lidocaine, swallowing or breathing difficulty was reported by two participants using lidocaine, and bruising/swelling was reported by six participants using lidocaine, 2 with articaine, 2 with mepivacaine, and 2 with prilocaine, respectively. Pain for >two weeks and under eye numbness were only reported with articaine by two participants each. Vision alteration was reported by two participants using articaine and 2 using other local anesthetic agents mentioned (Table 2). 

**Table 2:** Adverse effects reported by participants with different local anesthetic agents

S. No	Adverse effects	Local anesthetic agents					Total
		Lidocaine	Articaine	Mepivacaine	Prilocaine	Others	
1.	Vision alteration		2			2	4
2.	Under eye numbness		2				2
3.	Pain >two weeks		2				2
4.	Bruising/swelling	6	2	2	2		12
5.	Swallowing/breathing difficulty	2					2
6.	Syncope	2					2
7.	Anxiety/palpitations	12	4				16
8.	Palsy	4	2				6
9.	Hematoma	4			2		6
10.	Trismus	8				2	10
11.	Neuropathy	2	4				6
12.	Paresthesia	15	11	2		2	30

Of 70 subjects among 98 who reported adverse effects, no change in their practice was reported after encountering adverse effects. 17 stopped using the local anesthetic agents that caused adverse effects for the procedure where it was used, whereas six dentists completely stopped using that agent. Other five dental practitioners adopted other ways after adverse effects including stopping its use for the IANB, using mandibular block, and changing the needle gauge. On asking for any further information concerning these adverse effects, it was based on the change in technique than the LA and awareness about electric shock during anesthesia. For paresthesia, the majority were seen in subjects with epilepsy and were temporary with resolved within one year including lingual paresthesia with articaine.

On assessing the perception of dentists for the use of a local anesthetic agent, it was seen that the majority of dentists who participated in this study used country guidelines according to 25% (n=200) participants for the choice of LA followed by professional courses by 19.5% (n=156) participants, dental degree by 16.62% (n=133) participants, self-research by 15.5% (n=124) participants, colleagues suggestion by 13.87% (n=111) participants, experience by 10% (n=8) participants, mentors suggestion by 7.25% (n=58) participants, manufacturer’s instructions by 0.55 (n=4) participants, and evidence-based literature by 0.25% (n=2) participants (Table 3).

**Table 3:** Factors governing the choice of local anesthetic agents in study participants

S. No	Characteristics	Percentage (%)	Number (n=800)
1.	Manufacturer’s instructions	0.5	4
2.	Experience	100	8
3.	Evidence-based literature	0.25	2
4.	Mentors	7.25	58
5.	Colleagues	13.87	111
6.	Self-research	15.5	124
7.	Dental degree	16.62	133
8.	Professional courses	19.5	156
9.	Country guidelines	25	200
10.	Others	0.5	4

## Discussion

The majority of the study respondents agreed that they used articaine during their dental practice. These results were comparable to the findings of Yapp et al. in 2012, where a comparable proportion of dentists used articaine in their practice [6]. However, in the present study, 43% of respondents denied using it for the IANB, which was much higher compared to that reported by Yapp et al., where only 10% refrained from using articaine for the IANB. For its use in pediatric subjects, a previous study by Ezzeldin et al. in 2020 reported better efficacy of articaine compared to lidocaine with fewer adverse effects [7]. However, literature data assessing its use in pediatric subjects are scarce and need further assessment. Similarly, fewer data are available for the use of articaine in pregnant females [6,7,8].

The results of the present study showed that 60% (n = 480) participants used articaine in their practice, followed by lidocaine, which was used by 33% (n = 264) dental practitioners, mepivacaine by 2% (n = 16) participants, prilocaine by 1% (n = 8) dental practitioners, and other local anesthetics by 4% (n = 32) participants, respectively. Concerning the safety and efficacy of articaine use, 43% used it for all procedures except for the IANB, whereas 20% used it for all procedures, including IANB. These results were consistent with the previous studies of Tong et al. [8] in 2018 and Tsang et al. [9] in 2017, where authors reported a similar proportion of dental practitioners using different local anesthetic agents in their studies as of the present study.

The results of the present study also showed that various dental practitioners did not use them in various other conditions, including absolute contraindications, pediatric subjects, and pregnant females. Forty-three percent of participants considered articaine to be more efficacious and safer compared to lidocaine, 34% considered it less safe but more efficacious than lidocaine, and 19% found it to be equally efficacious and safe as lidocaine. Four participants considered articaine unsafe to be used as a local anesthetic. These findings were in line with the previous results of Carr et al. [10] and Soysa et al. [11], where authors reported similar reasons for not using articaine in their dental practice.

In the present study, 12.25% (n = 98) of the participants reported one or more adverse effects following the use of local anesthesia. The most common adverse effect was paresthesia, reported by 30 participants: 11 cases were reported with lidocaine, 15 with articaine, and 2 each with mepivacaine and other local anesthetics. This was in agreement with the previous studies of Yapp et al. in 2011, where the authors reported paresthesia in one in 785,000. The scarce data available can be attributed to a lower incidence, which leads to fewer studies available, as also reported by Gaffen et al. [12] in 2009, where authors reported less evidence of paresthesia following the IANB. Also, the data are controversial regarding the cause of paresthesia after the IANB, which is commonly seen after lingual nerve paresthesia, as suggested by Garisto et al. [13] in 2010. The possible reasons reported by Yapp et al. [14] in 2011 were LA toxicity, fascicular pattern, hematoma formation, and needle trauma.

The limitations associated with the present study are the authenticity of the participants who filled out the study questionnaire, which makes the data less reliable. Also, the cost of articaine is higher than that of lidocaine, which was not focused on in the present study. However, in Indian practice, cost remains a common factor to be considered. Also, considering the study by Safdar et al. [15] in 2016, despite survey studies being a convenient, time-effective, and cost-effective method, unclear responses, biases, and non-clarified results are usually associated with survey assessments, raising questions about their reliability.

## Conclusions

The present study discussed the effectiveness of safety and various measures about articaine. The results of the present study also showed that various dental practitioners did not use them in various other conditions, including absolute contraindications, pediatric subjects, and pregnant females. Considering its limitations, the study pointed to the conclusion that despite the reported efficacy and safety of articaine as a local anesthetic for all dental procedures, many dental practitioners refrain from using articaine, especially for IANBs. These data depict a difference between current research evidence and reported clinical practice.
